# Data and tools to integrate climate and environmental information into public health

**DOI:** 10.1186/s40249-018-0501-9

**Published:** 2018-11-29

**Authors:** Pietro Ceccato, Bernadette Ramirez, Tawanda Manyangadze, Paul Gwakisa, Madeleine C. Thomson

**Affiliations:** 1 The International Research Institute for Climate and Society, The Earth Institute, Columbia University, 61 Route 9W, Lamont-Doherty, Palisades, NY 10964 USA; 2 The Special Programme for Research and Training in Tropical Diseases (TDR), World Health Organization, Geneva, Switzerland; 3 School of Nursing and Public Health, Department of Public Health, College of health Sciences, University of KwaZulu-Natal, P. Bag, 1020 Bindura, Zimbabwe; 4 South Africa and Geography Department, Faculty of Sciences, Bindura University of Science Education, P. Bag, 1020 Bindura, Zimbabwe; 5 Nelson Mandela African Institution of Science and Technology, School of Life Sciences and Bioengineering, P.O. Box 447, Arusha, Tanzania; 6 Present address: Sokoine University of Agriculture, P.O. Box 3019, Morogoro, Tanzania

**Keywords:** Climate and environmental information, Data, Access, Tools, Geographical information system, Malaria, Schistosomiasis, Trypanosomiasis

## Abstract

**Background:**

During the last 30 years, the development of geographical information systems and satellites for Earth observation has made important progress in the monitoring of the weather, climate, environmental and anthropogenic factors that influence the reduction or the reemergence of vector-borne diseases. Analyses resulting from the combination of geographical information systems (GIS) and remote sensing have improved knowledge of climatic, environmental, and biodiversity factors influencing vector-borne diseases (VBDs) such as malaria, visceral leishmaniasis, dengue, Rift Valley fever, schistosomiasis, Chagas disease and leptospirosis. These knowledge and products developed using remotely sensed data helped and continue to help decision makers to better allocate limited resources in the fight against VBDs.

**Main body:**

Because VBDs are linked to climate and environment, we present here our experience during the last four years working with the projects under the, World Health Organization (WHO)/ The Special Programme for Research and Training in Tropical Diseases (TDR)-International Development Research Centre (IDRC) Research Initiative on VBDs and Climate Change to integrate climate and environmental information into research and decision-making processes. The following sections present the methodology we have developed, which uses remote sensing to monitor climate variability, environmental conditions, and their impacts on the dynamics of infectious diseases. We then show how remotely sensed data can be accessed and evaluated and how they can be integrated into research and decision-making processes for mapping risks, and creating Early Warning Systems, using two examples from the WHO TDR projects based on schistosomiasis analysis in South Africa and Trypanosomiasis in Tanzania.

**Conclusions:**

The tools presented in this article have been successfully used by the projects under the WHO/TDR-IDRC Research Initiative on VBDs and Climate Change. Combined with capacity building, they are an important piece of work which can significantly contribute to the goals of WHO Global Vector Control Response and to the Sustainable Development Goals especially those on health and climate action.

**Electronic supplementary material:**

The online version of this article (10.1186/s40249-018-0501-9) contains supplementary material, which is available to authorized users.

## Multilingual abstracts

Please see Additional file 1 for translations of the abstract into the five official working languages of the United Nations.

## Background

During the last 30 years, the development of geographical information systems (GIS) and satellites for Earth observation has made important progress that had made it possible to monitor weather, climate, environmental and anthropogenic factors that influence the reduction or the reemergence of vector-borne diseases (VBDs). Analyses resulting from the combination of GIS and remote sensing have improved knowledge of climatic, environmental, and biodiversity factors [1, 2], influencing vector- borne diseases such as malaria [3, 4], visceral leishmaniasis (VL) [5–7], dengue [8–10], Rift Valley fever [11, 12], schistosomiasis [13–16], Chagas disease [17, 18], and leptospirosis [19, 20]. This knowledge and products, developed using remotely sensed data, helped and continue to help decision makers to better allocate limited resources in the fight against VBDs. Because VBDs are linked to climate and environment, we present here our experience during the last 4 years working with the projects under the World Health Organization (WHO)/ The Special Programme for Research and Training in Tropical Diseases (TDR)-International Development Research Centre (IDRC) Research Initiative on VBDs and Climate Change [21, 22] to integrate climate and environmental information into research and decision-making processes.

The following sections present the methodology we have developed, which uses remote sensing to monitor climate variability, environmental conditions, and their impacts on the dynamics of infectious diseases. We then show how remotely sensed data can be accessed and evaluated and how they can be integrated into research and decision-making processes for mapping risks, and creating Early Warning Systems (EWS), using two examples from the WHO TDR projects [21] based on schistosomiasis analysis in South Africa and Trypanosomiasis in Tanzania.

## Climate and environmental factors: How do they help?

To date, much of the debate has centered on attribution of past changes in disease rates to climate change and the use of scenario-based models to project future changes in risk for specific diseases (e.g., for schistosomiasis [23–25]). Although these can give useful indications, the unavoidable uncertainty in such analyses, as well as contingency on other socioeconomic and public health determinants in the past or future, limit their utility as decision-support tools. The output predictive models should also be validated against field observations as argued by reference [26] to realize their usefulness in community health and climate change decision making process especially at the local level in Africa [15]. For operational health agencies, the most pressing need is the strengthening of current disease control efforts to bring down current disease rates and manage short-term climate risks, which will, in turn, increase resilience to long-term climate change. The WHO and partner agencies are working through a range of programs to (1) ensure political support and financial investment in preventive and curative interventions to bring down current disease burdens; (2) promote a comprehensive approach to climate risk management; (3) support applied research, through definition of global and regional research agendas and targeted research initiatives on priority diseases and population groups [27].

In this context, the International Research Institute for Climate and Society (IRI) develops research and capacity building together with researchers, policy/decision makers, public health practitioners, and communities in lower middle income disease endemic countries to enable access and use of climate services to first understand the mechanisms driving changes in transmission of diseases. We first try to understand the relationship between diseases and climate by creating spatial and temporal stratification of the diseases and population at risk (i.e. risk mapping) [28, 29]. If a relationship exists between the diseases and climate, we estimate the seasonality of the disease and timing of intervention. We then develop frameworks for EWS to monitor in real time and forecast the risks of diseases transmission based on climate and environmental factors. Finally, once decision makers have put in place control measures to mitigate the problem, climate variability is considered to assess the efficacy of control measures (i.e. evaluation stage of mitigation measures). For example, if malaria control intervention scale-up follows an unusually wet and warm baseline period and malaria incidence declines following interventions (during a drier and or cooler period), it may be tempting to attribute all of the decline in malaria outcomes to the investments in malaria control. Correct attribution is important. As climate varies naturally over time, it is likely that the situation will at some point reverse, resulting in an increase in climate suitability for transmission risk. If climate is not accounted for, then higher malaria cases observed may be inappropriately attributed to program failure [30].

### Early warning system

The WHO has developed a framework for creating an EWS for malaria [31]. The framework is composed of four components:

1. Vulnerability assessment, including the assessment of current control measures, any problems related to resistance developed by the mosquitoes or the plasmodium parasites, socioeconomic factors, such as migration of population, and so on.
2. Climate forecasting, allowing for forecasting, 3–6 months in advance, of the probability of an increase in precipitation or in temperature, weather conditions that may lead to an increase in risk for an outbreak of malaria.
3. Monitoring of climate and environmental factors, including monitoring of precipitation, temperature, and the presence of vegetation or water bodies that would influence the development of mosquitoes.
4. Case surveillance: Monitoring of malaria cases is either performed at the hospital level or by health workers by visiting community as active surveillance. The data are then managed at the central level by the Ministry of Health.

### Evaluation of control measures

The President Malaria Initiative (PMI) uses the Roll Back Malaria (RBM) partnership–approved methodology to evaluate whether the deployed interventions have had an impact on malaria morbidity and mortality. The methodology requires consideration of contextual (potentially confounding) factors that affect the epidemiology of malaria when using all-cause mortality as the measure of impact [30]. These factors include increases in household income, better drug and mosquito net distributions, improvements in living conditions, and so on. Although the RBM methodology provides guidance on how to consider certain confounding factors when determining their potential impact on mortality, the effect of climate on malaria prevalence, and therefore mortality, is much less clear.

In order to conduct the analysis for the above three components, availability of decision-relevant climate and environmental information about the past, recent trends, current conditions, likely future trajectories, and associated impacts is a prerequisite for climate-informed decision-making [30].

## Accessing quality data through earth observations

When working on VBDs, decision makers and researchers often face a lack of quality data required for optimal targeting of the intervention and surveillance. The results/decisions are critical as they impact on the lives of many people: “Bad data create bad policies” [32].

Climate data and information—whether station- or satellite-generated—can increasingly be accessed freely online [33, 34]. Station data (most commonly observations of rainfall and minimum and maximum temperatures) can typically be obtained from a country’s National Meteorological and Hydrological Service (NMHS). Depending on the quality control processes performed by the NMHS, these data may be of varying quality. However, access to station data (especially daily) is not always readily available especially in Africa. Some of the station data provided by the NMHS are freely available through the Global Telecommunication System but often lack the spatial coverage needed. Weather and environmental monitoring satellite sensors gather data that are continuously archived and cover large areas of the globe. In order for decision makers to access, visualize, or manipulate these data, they must first be converted to relevant information and then shared through an appropriate interface. In many cases, the raw data may be free, but processing the data appropriately requires technical skills and not all interfaces allow free access to their archived data. Sources for satellite-generated climate data are varied, and a selection is provided below. The following are likely to be the most useful of the freely available satellite-based estimates. They all differ in the strengths and weaknesses and the best choice for one situation may not be the best choice elsewhere.

### Precipitation

No satellite yet exists that can reliably identify rainfall and accurately estimate the rainfall rate in all circumstances. Satellite can see the clouds from above that we see from below, but cloud presence is not a good indicator of rainfall. Not all clouds produce rain, and rainfall intensity varies from place to place beneath those clouds that are generating rain. Using a variety of sensors, it is possible to distinguish raining cloud from non-raining cloud by estimating:

- *Cloud-top temperatures*: deep convective clouds have cold, high tops, and so areas of deep convection show up as low temperatures. This method of identification works best in the tropics and in the mid-latitude summer months when convective rainfall may predominate. However, other types of rainfall may go unidentified because they do not form from cold clouds, and there may be false detection of rainfall from non-raining cold clouds. Such errors may be substantial in regions near the coast or in mountainous areas. Although estimates of rainfall from cloud-top temperatures have good spatial coverage, high temporal resolution and frequent updates (every 15–30 min), the accuracy is often poor.
- *Cloud thickness*: rather than using the temperature of the cloud top as a proxy for the intensity of deep convection, the amount of water and ice in the cloud can be estimated by measuring the amount of scattered microwave radiation. These methods offer a more accurate rainfall estimate, but have coarse spatial resolution and are updated only twice a day. Currently, the estimates are least accurate over the land, where, unfortunately, the information is needed most.

Techniques are being developed to take advantage of the better accuracy of microwave sensors and the better spatial and temporal coverage of infrared sensors by optimally combining the two products. A variety of monitoring products is becoming available using different ways of combining the products as follows:

- The *Global Precipitation Climatology Project* (GPCP) combines satellite and station data. The monthly data extend from 1979 onwards, while the daily product is from 1996 to present.^1^ The product is available at 250 km spatial resolution in the IRI Data Library at: http://iridl.ldeo.columbia.edu/SOURCES/.NASA/.GPCP/.V2p3/.CDR/.precip/
- The *Climate Prediction Center (CPC) Merged Analysis of Precipitation* (CMAP) combines satellite and station data.^2^ This product is very similar to the GPCP but has some differences due to different algorithms used to estimate precipitation. The product is available at 250 km spatial resolution in the IRI Data Library at: http://iridl.ldeo.columbia.edu/SOURCES/.NOAA/.NCEP/.CPC/.Merged_Analysis/.monthly/.latest/.ver2/.prcp_est/
- The *CPC MORPHing technique* (CMORPH) provides global precipitation estimates at very high spatial (25 km) and temporal (3 h) resolutions.^3^ This product is suitable for real-time monitoring of rainfall, provided a long history is not required, as data are only available from January 1998. The product is available in the IRI Data Library at: http://iridl.ldeo.columbia.edu/SOURCES/.NOAA/.NCEP/.CPC/.CMORPH/
- The *Tropical Rainfall Measurement Mission* (TRMM) provides estimates of precipitation in the tropics. Monthly aggregates improve the quality of the data. They are available from January 1998 to May 31 2015. The product is of good quality if high spatial (25 km) detail is required and real-time information is not critical.^4^ The product is available in the IRI Data Library at: http://iridl.ldeo.columbia.edu/SOURCES/.NASA/.GES-DAAC/.TRMM_L3/.TRMM_3B42/.v7/.daily/.precipitation/
  - The *Global Precipitation Measurement* (GPM) provides estimates of precipitation globally. They are available from March 2014 to present [35]. The GPM is an extension of the TRMM rain-sensing package.^5^ The product is available at: https://gpm1.gesdisc.eosdis.nasa.gov/data/GPM_L3/GPM_3IMERGDF.05/
  - The *African Rainfall Estimate* (RFE) combines satellite and station data specifically for Africa. The data are available from 1995 and are useful for high spatial resolution (11 km).^6^ The product is available in the IRI Data Library at: https://iridl.ldeo.columbia.edu/SOURCES/.NOAA/.NCEP/.CPC/.FEWS/.Africa/.DAILY/.RFEv2/.est_prcp/
  - The *Enhancing National Climate Services* (ENACTS) program combines all available rain gauge data from the NMHSs of Ethiopia, Gambia, Ghana Madagascar, Mali, Rwanda, Tanzania, Kenya and Zambia, with satellite data for the last 30 years at high spatial resolution.^7^ Because the ENACTS rainfall products includes many more observations than are available in the global products described above the program generates the best quality data sets available at the national level. The programme is continuing to expand to other countries in Africa [36]. The products are available at the Met Services in each country where ENACTS has been installed.
  - *Climate Hazards Group Infrared Precipitation with Station* (CHIRPS) data are produced by the University of California, Santa Barbara, using a similar technique developed to create the ENACTS data but using fewer rain gauges.^8^ The product at 5 km spatial resolution is available in the IRI Data Library at: https://iridl.ldeo.columbia.edu/SOURCES/.UCSB/.CHIRPS/.v2p0/.monthly/.global/.precipitation/

### Temperature

Air temperature is commonly obtained from synoptic measurements in weather stations measured at 2-m high. In Africa, the spatial distribution of weather stations is often limited and the dissemination of temperature data is variable, therefore limiting their use for real-time applications. Compensation for this paucity of information may be obtained by using satellite-based methods. The estimation of near-surface air temperature (Ta) is useful for a wide range of applications in health. It affects the transmission of malaria [37] in the highlands of East Africa. However, the derivation of Ta from the land-surface temperature (LST) derived from satellite is far from straightforward. In cloudless conditions, the satellites can measure the temperature of Earth’s surface, but the surface temperature is not necessarily a good indication of the air temperature. Although night-time satellite products provide reasonable estimates of minimum temperatures, maximum temperature estimates are problematic [38].

Studies have shown that it is possible to retrieve high- resolution Ta data from the moderate-resolution imaging spectroradiometer (MODIS) Ts products over different ecosystems in Africa [38–40].

For temperature-based data, the following data sets are recommended:

- Land-surface temperature (LST) from MODIS provides land-surface temperature estimates. The data are available from July 2002 for Africa and from March 2000 for South America at a spatial resolution of 1 km. Separate estimates for daytime and night-time temperatures are available. Maximum and minimum air temperature estimates can be derived from the land-surface temperatures [39]. The products are available in the IRI Data Library at: https://iridl.ldeo.columbia.edu/SOURCES/.USGS/.LandDAAC/.MODIS/.1km/.8day/.version_005/

### Vegetation

Remote sensing can be used to distinguish vegetated areas from bare soils and other surface covers. Various vegetative properties can be derived from indices such as the Normalized Difference Vegetation Index (NDVI), including but not limited to leaf area index, biomass, greenness, and chlorophyll. However, quantitative analyses are highly sensitive to the context of the study location, and relationships should be assessed prudently.

Practitioners can access data on vegetation cover through the following sources:

- *Global NDVI* is available from 1981 to 2004. The data set has been shown to be valid in representing vegetation patterns in certain regions (but not everywhere) and should be used with caution [41]. The product is available in the IRI Data Library at: https://iridl.ldeo.columbia.edu/SOURCES/.UMD/.GLCF/.GIMMS/.NDVIg/.global/.sat/
- *Terra MODIS NDVI* and Enhanced Vegetation Index (EVI) are available for 16-day periods from April 2000 at 250-m resolution. The NDVI is an updated extension to the Global NDVI. The EVI is another index used to estimate vegetation that can complement the NDVI [42]. The products are available for different regions of the world in the IRI Data Library at: https://iridl.ldeo.columbia.edu/SOURCES/.USGS/.LandDAAC/.MODIS/.version_006/

### Water bodies and inundation products

Using LANDSAT images at 30-m spatial resolution, it is possible to map small water bodies where mosquitoes will breed and transmit diseases such as malaria, dengue fever, chikungunya, West Nile fever and where snails breed transmitting schistosomiasis [16, 43]. By combining the middle-infrared channel (which is sensitive to water absorption), the near-infrared channel (which is sensitive to bare soil and vegetation canopy), and the red channel (which is sensitive to chlorophyll absorption), it is possible to map water bodies in blue, vegetation in green, and bare soils in brown [44]. Using a technique developed by Pekel et al. [44], it is possible to map the water bodies by transforming the red–green–blue color space (represented by the middle infrared, near- infrared and red channels) into a hue–saturation–value space that decouples chromaticity and luminance. Global map of water at high special resolution based on LANDSAT for the last 30 years are now made available on-line at: https://global-surface-water.appspot.com/ [45].

Global maps of inundated area fraction are also derived at 25-km scale from remote sensing observations from multiple satellite sources [46], focusing on data sets from active/passive microwave instruments (European Remote Sensing scatterometer, QuikSCAT, Special Sensor Microwave/Imager, and Advanced Microwave Scanning Radiometer). Those products are used to map flood events and their impacts on malaria and leishmaniasis in South Sudan [7].

Practitioners can access data on water bodies through the following sources:

- *Terra MODIS middle-infrared, near-infrared, and red reflectances* are available for 16-day periods from April 2000 onward at 250-m resolution. The products are available in the IRI Data Library at: https://iridl.ldeo.columbia.edu/SOURCES/.USGS/.LandDAAC/.MODIS/.version_006/
- *LANDSAT middle-infrared, near-infrared, and red reflectances* are available every 16 days at 30-m spatial resolution. The products can be accessed using Google Earth Engine (example: https://code.earthengine.google.com/b4a5ea6ac0a8fe5520ec039c98abaff5)
- *Inundation fraction* products are available for daily, 6-day, and 10-day periods for the entire globe at 25-km spatial resolution [47]. The products are available through the IRI Data Library at: https://iridl.ldeo.columbia.edu/SOURCES/.NASA/.JPL/.wetlands/

## Data accessibility

Over the past 30 years, the field of remote sensing has grown to include numerous national, intergovernmental, and private organizations that freely provide user-friendly high spatial and temporal resolution data sets. However, the ease of access should not be mistaken for ease of analysis as the data sets are still complex and require complex evaluation, especially when applied to decision-making.

The IRI has developed various tools and provided capacity building to improve data accessibility and analysis for decision makers and interdisciplinary researchers alike. A Climate Data Library was built as an integrated knowledge system to support the use of climate and environmental information in climate-sensitive health decision-making. Initiated as an aid to climate scientists to do exploratory data analysis, it has expanded to provide a platform for transdisciplinary researchers focused on topics related to climate impacts on society.

### IRI data library

The IRI Climate Data Library is organized as a collection of both locally held and remotely held data sets, designed to make the data more accessible for the library’s users. Data sets in the library come from many different sources in many different formats [33].

The IRI Climate Data Library can be used via two distinct mechanisms that are designed to serve different communities. Expert Mode serves the needs of operational practitioners and researchers that have an in-depth knowledge of the functionality of the system and are able to customize it to their own specific needs (see: http://iridl.ldeo.columbia.edu/SOURCES/#info). The Data Library programming language (Ingrid) can be used by advanced users to develop custom functions and perform tailored analyses (see: http://iridl.ldeo.columbia.edu/dochelp/StatTutorial/index.html). Expert Mode allows users with programming skills a very extensive level of personalized functionality. Online tutorials, examples, and function definitions are part of the Data Library [33].

#### Map rooms

In contrast to Expert Mode, the Map Rooms (see: http://iridl.ldeo.columbia.edu/maproom/) provide easy access to point-and- click map-based user interfaces that are built on Data Library infrastructure. The Map Rooms are the result of collaborative negotiations around information needs and make specific data and products for a region or time period available for a specific purpose to specific users and decision makers. The data and maps in these Map Rooms are available for quick and easy download to the user’s desktop.

#### IRI climate data library archives and near-real-time updates

Global climate observations by ground stations, satellites, and modeled estimates of climatic conditions compose the vast majority of the Data Library’s data archive. An extensive menu of maps and analysis used to monitor current global and regional climate, as well as historical data, are available from a wide range of sources including National Aeronautics and Space Administration (NASA), National Oceanic and Atmospheric Administration (NOAA), Climatic Research Unit University of East Anglia (CRU- UEA), World Meteorological Organization (WMO), European Centre for Medium-Range Weather Forecasts (ECMWF), Goddard Institute for Space Studies (GISS, and so on [32]. From the Map Rooms, it is possible to readily access and download the publicly available data sets being viewed, including station, atmospheric, and oceanic observations and analyses, model-based analyses, and forecasts, as well as land-surface and vegetation information.

The near-real-time data sets are updated by automated software that retrieves the data as soon as it is available on the originating site. For instance, MODIS satellite data will be available in the IRI Climate Data Library within a day after processing is complete at the NASA data center.

#### Downloading data library data and products

A Data Library user can download both images and data onto a desktop workstation. Data can be downloaded in standard ASCII and binary formats, Excel and R tabular formats, GIS formats, netCDF files, and directly to application software (such as GrADS and MATLAB®) that support the OPeNDAP data transfer protocol [48]. Over the last decade, OPeNDAP has emerged as a community standard for machine-to-machine data access and transfer and is widely used where data sharing is involved, for example, with the climate change scenarios developed as part of the Coupled Model Intercomparison Project for the Intergovernmental Panel on Climate Change [49].

Images, including maps, produced in the Data Library can be delivered to the user’s desktop in standard graphics formats like PostScript, JPEG, and PDF. The maps can also be made available in WMS, KML, and GIS formats that feed directly into applications such as Google Earth, Google Maps, or ArcGIS. Any analysis or data download done by the user is represented in a URL that can be saved to the user’s desktop. This URL can be shared with collaborators to repeat the analysis. The URL can be incorporated into a script that is run periodically when either environmental or public health data sets are updated.

The IRI Data Library has enabled decision makers to have fast and easy access to the different Earth Observation products mentioned in the section “Improving Data Quality and Accessibility” and to analyze the data to understand the seasonality and trends of climate in relation to health.

### Google earth engine

Google Earth Engine (GEE) is a cloud-based platform for planetary-scale geospatial analysis that brings Google’s massive computational capabilities to bear on a variety of high-impact societal issues including deforestation, drought, disaster, disease, food security, water management, climate monitoring and environmental protection. It is unique in the field as an integrated platform designed to empower not only traditional remote sensing scientists, but also a much wider audience that lacks the technical capacity needed to utilize traditional supercomputers or large-scale commodity cloud computing resources [34].

GEE makes it easy to access high-performance computing resources for processing very large geospatial datasets, without having to suffer the IT pains currently surrounding either. Additionally, and unlike most supercomputing centers, Earth Engine is also designed to help researchers easily disseminate their results to other researchers, policy makers, NGOs, field workers, and even the general public. Once an algorithm has been developed on Earth Engine, users can produce systematic data products or deploy interactive applications backed by Earth Engine’s resources, without needing to be an expert in application development, web programming or HTML.

## Integration of climate and environmental data within WHO/TDR projects

During the five years of the WHO/TDR-IDRC Research Initiative on VBDs and Climate Change project [21], we have been collaborating with the five teams to provide training on how to integrate the climate and environmental data using the tools and methodologies described above. More in depth descriptions of the five projects that encompassed malaria, trypanosomiasis, Rift Valley Fever and schistosomiasis are provided in this special issue journal and additional peer review publications such as in reference [50]. Here we present succinctly how climate and environmental data from the IRI Data Library and Google Earth Engine were integrated into VBD.

### Schistosomiasis

In the uMkhanyakude district of South Africa, Manyandadze et al. [16] discovered that the snails carrying and transmitting schistosomiasis are most likely to be found where there is slow moving surface water with slightly higher-than-normal temperatures. But the snails can also hibernate when the pools get dry and then repopulate during and after the rainy season. Such pools are often where people enter and then come into contact with the parasite.

Using a new model, Manyandadze tested variables derived from the IRI Data Library such as air temperature, rainfall, water velocity (as estimated by the slope of ground) and soil pH to try to predict where the snails would be found, and then compared those findings with sampling of snails in the field. They found that the best predictor of where snails are present is a measure called the Normalized Difference Water Index (NDWI), which estimates the presence of surface water bodies based on satellite data and a mathematical formula.

**Fig. 1:**
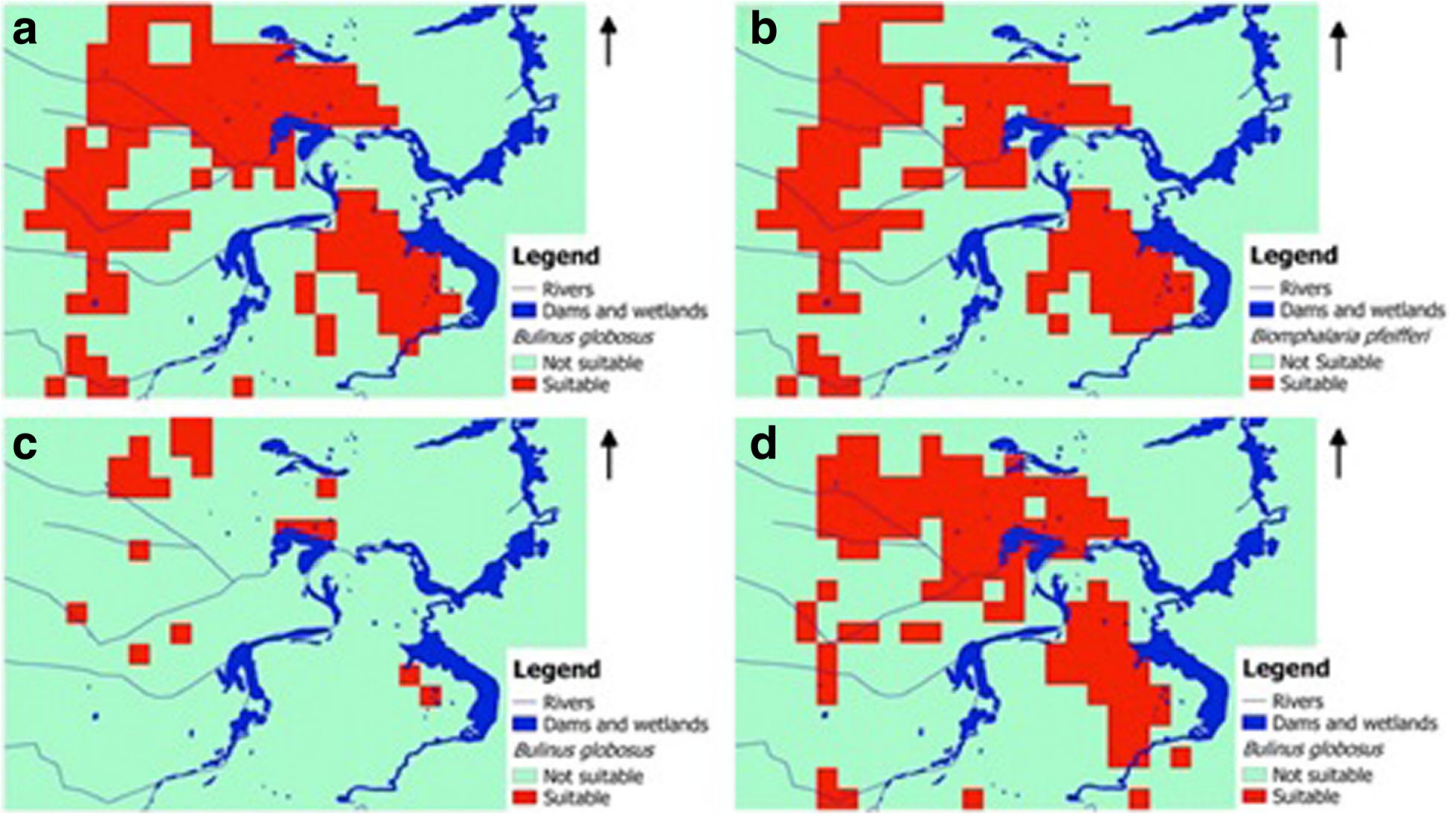
Seasonal suitable and not suitable habitats for two snail species in Ndumo area of uMkhanyakude district, South Africa based on Maxent model using climatic and environmental factors: (**a**) *Bulinus globosus* in cold/dry season (June to August). (**b**) *Biomphalaria pfeifferei* in cold/dry season (June to August). (**c**) *Bulinus globosus* in hot/dry season (September to November). (**d**) *Bulinus globosus* in post rainy season (March to May) (adapted from Manyangadze et al. 2016 [16])

The mapping techniques are particularly useful in areas with distinct dry and wet seasons, where temporary bodies of water may form in some years but not others, and sometimes in different locations. The maps (Fig. 1) produced by the model can help health workers narrow-in on where the risk of schistosomiasis may be high. With that information, they can take actions such as stockpiling medications that interrupt the parasite’s cycle, controlling snail populations and launching awareness campaigns. Without such a model, much more time and resources must be spent to send surveyors to identify areas of probable risk.

### Trypanosomiasis

**Fig. 2:**
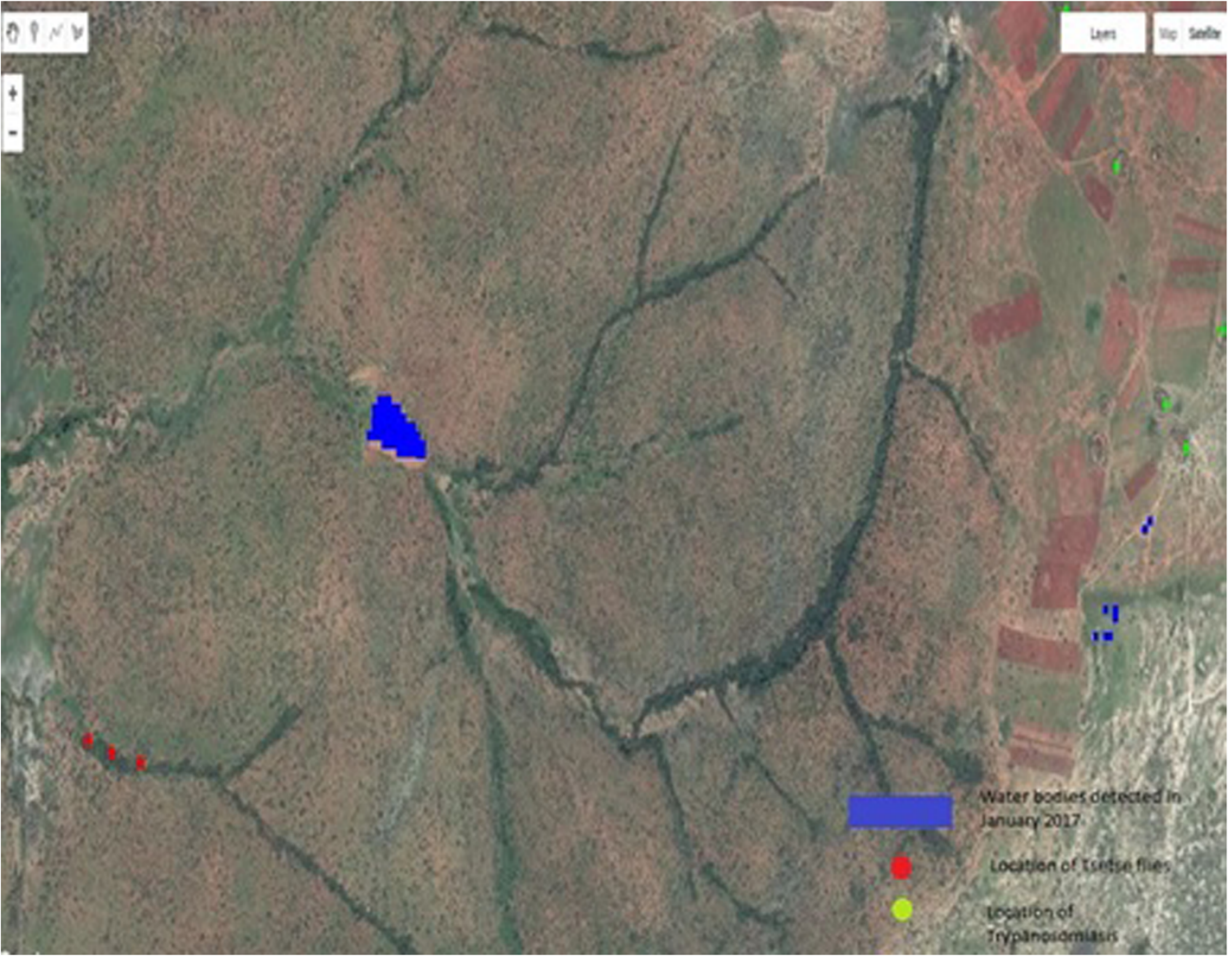
Very high spatial resolution image with location of water bodies detected in January 2017 (blue color), location of tsetse flies (red dots) and location of trypanosomiasis (green dots)

Tackling Sleeping Sickness in Maasai Communities is one of the five projects supported by the WHO/TDR-IDRC Research Initiative on VBDs and Climate Change [21]. Using GEE, we developed applications for Climate/Environment/Health allowing researchers and the Maasai community to access global precipitation datasets, temperatures, vegetation and water bodies at high spatial resolution from LANDSAT and Sentinel 2, floods from Sentinel 1 (Radar Systems) and very high spatial resolution datasets (QuickBird, Ikonos). It is now possible to integrate algorithms to access satellite images, create products and integrate them with population datasets, infrastructure from high spatial resolution images and disease data (e.g., on trypanosomiasis), see Fig. 2.

**Fig. 3:**
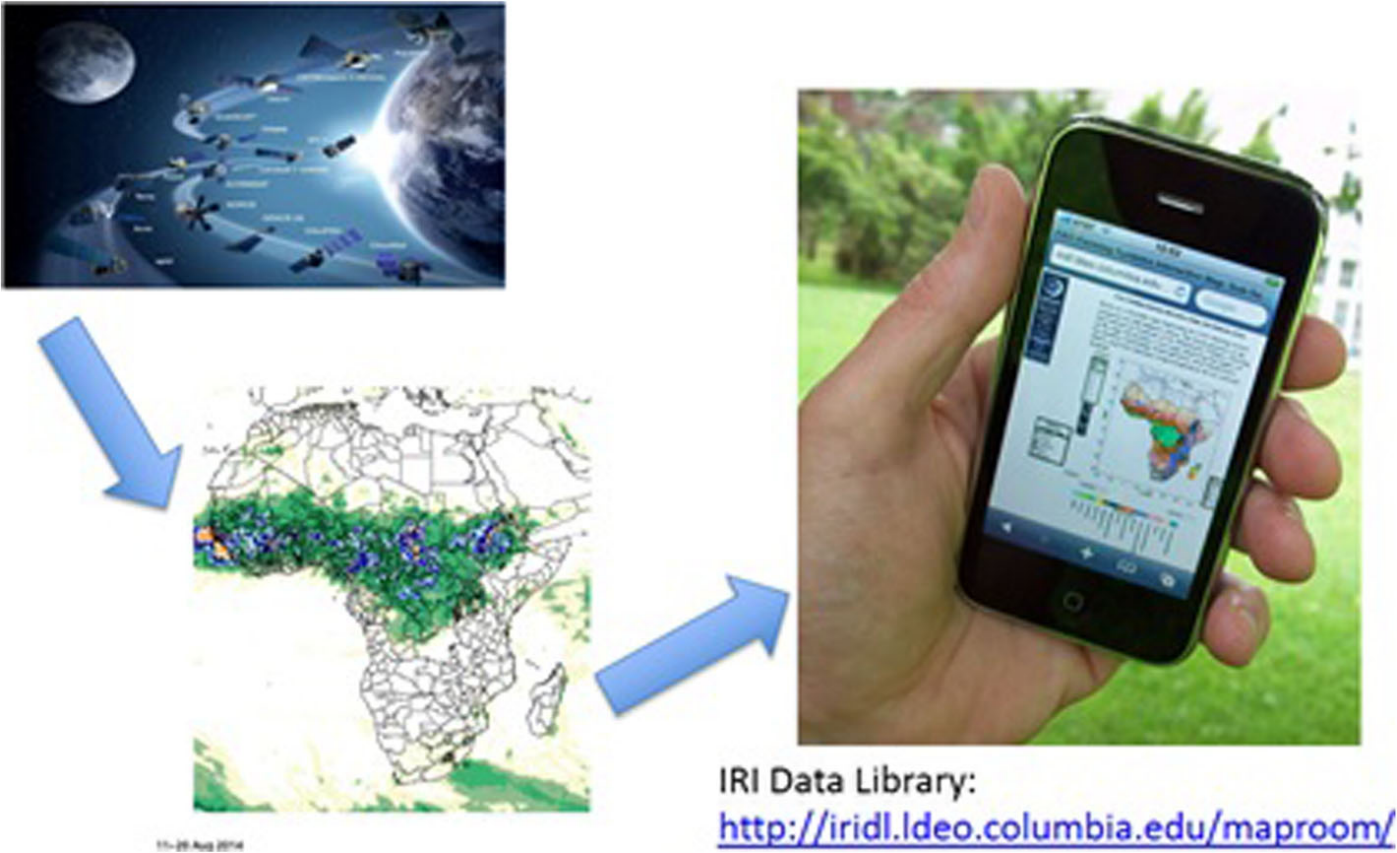
Dissemination of climate data derived from earth observation to local communities through the IRI Data Library and Google Earth Engine

**Fig. 4:**
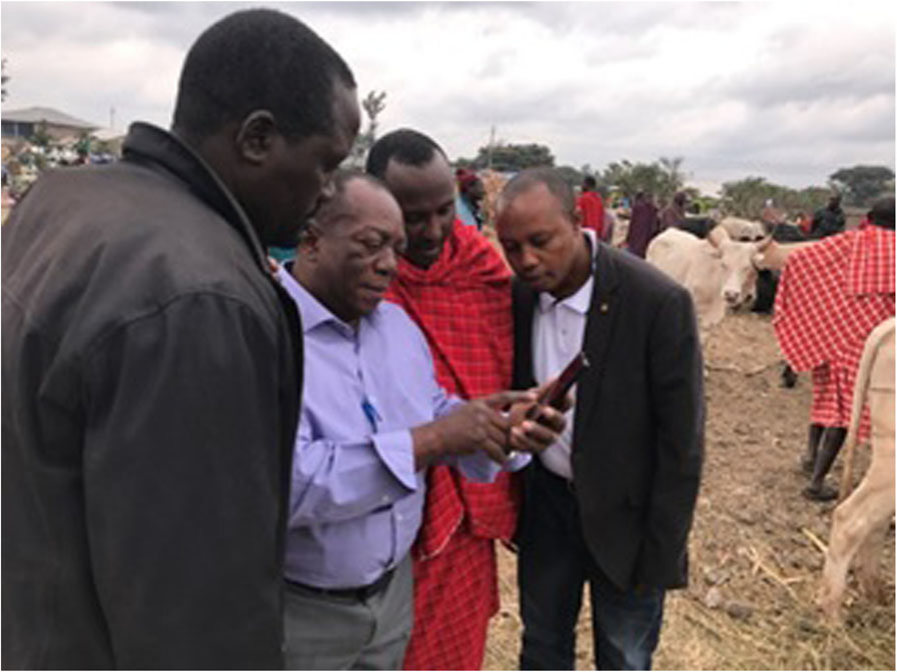
Demonstration of the climate, environmental and trypanosomiasis interface on smartphone to the Maasai community in Arusha, Republic of Tanzania (photo used with permission from Paul Gwakisa)

In addition to using GEE, we developed an application on smartphone that is used to access and analyze satellite images on precipitation, temperature, water bodies (based on LANDSAT images) and integration with local data on the presence of the tsetse flies and trypanosomiasis. This new smartphone application allowed users to access high spatial resolutions images and extract time-series analysis for mapping the risks of trypanosomiasis in Maasai villages in northern Tanzania (Figs. 3 & 4).

In addition to developing smartphone applications to integrate and analyse health data in conjunction with climate and information, we can develop smartphone applications to collect health data (geo-referenced with pictures of the environment and breeding sites). This application is based on the Open Data Kit (ODK) which is a free open-source set of tools which help organizations author, field, and manage mobile data collection solutions. ODK provides an out-of-the-box solution for user to:

- Build a data collection form or survey;
- Collect the data on a mobile device and send it to a server and;
- Aggregate the collected data on a server and extract it in useful formats.

Socio-economic and health surveys with GPS locations and images can be ingested by ODK and create decision support for clinicians for building multimedia-rich nature mapping tools.

## Conclusions

During the last 30 years, much progress has been made in incorporating remote sensing and GIS into decision processes that can help Ministries of Health and researchers in fighting vector-borne diseases. The examples provided in this article show how climatic and environmental factors can be monitored using remote sensing and integrated into decision-making process for mapping risks, creating EWS, and evaluating the impacts of control measures. Until recently, image and processing costs prevented local decision makers from implementing remote sensing decision-support systems on a large scale. More recently, computer processing, data storage facilities, and easy access to remotely sensed products have become available at low cost, and high spatial resolution images have become accessible free of charge. Processing tools are also being made available to the user community at no cost (e.g., IRI Data Library, Google Earth Engine). These developments have paved the way toward making countries more receptive to the implementation of remote sensing systems [32].

The tools presented in this article have been successfully used by the projects under the WHO/TDR-IDRC Research Initiative on VBDs and Climate Change. Combined with capacity building, they are an important piece of work which can significantly contribute to the goals of WHO Global Vector Control Response and to the Sustainable Development Goals (SDGs) especially those on health and climate action.

## Additional file

Additional file 1: Multilingual abstracts in the five official working 331 languages of the United Nations. (PDF 659 kb)

## Abbreviations

CHIRPS: Climate Hazards Group Infrared Precipitation with Station
CMORPH: CPC MORPHing technique
CPC: Climate Prediction Center
ENACTS: Enhancing National Climate Services
EWS: Early Warning Systems
GEE: Google Earth Engine
GIS: Geographical information systems
GPCP: Global Precipitation Climatology Project
GPM: Global Precipitation Measurement
IDRC: International Development Research Centre
IRI: International Research Institute for Climate and Society
LST: Land-surface temperature
NDVI: Normalized Difference Vegetation Index
NDWI: Normalized Difference Water Index
NMHS: National Meteorological and Hydrological Service
PMI: President Malaria Initiative
RFE: African Rainfall Estimate
Ta: Near-surface air temperature
TRMM: Tropical Rainfall Measurement Mission
VBDs: Vector-borne diseases
VL: Visceral leishmaniasis

## Footnotes

1 GPCP provides global monthly 2.5° and daily 1° rainfall estimates

2 CMAP provides products at a spatial resolution of 2.5° with 5-day and monthly aggregations since 1979.

3 CMORPH provides precipitation analyses at 8-km spatial resolution and 30-min temporal resolution.

4 TRMM provides precipitation data at 0.25°.

5 GPM provides precipitation data at 0.1°.

6 RFE has an 11-km spatial resolution and a 10-day temporal resolution

7 ENACTS uses a spatial resolution of 10 km, and 10-day and monthly products are available for the last 30 years. The approach is now being considered for expansion into other countries in Kenya, Uganda, and West Africa

8 The CHIRPS data cover the African continent at 5 km on a daily basis for the last 30 years and at 5 km every 10 days and on a monthly basis for the entire globe. The data are available via the IRI Data Library at: http://iridl.ldeo.columbia.edu/SOURCES/.UCSB/.CHIRPS/.v1p8/
