# Supplementary material for: Data and tools to integrate climate and environmental information into public health
Source: Infect Dis Poverty. 2018 Nov 29;7:126. doi: 10.1186/s40249-018-0501-9 (PMC6292116; doi:10.1186/s40249-018-0501-9)
Supplement: Supplementary file 1 — Multilingual abstracts in the five official working 331 languages of the United Nations. (PDF 659 kb) [file 40249_2018_501_MOESM1_ESM.pdf]

Translation of the abstract into the six official working languages of the United Nations

- المنهجيات والأدوات الضرورية لإدراج المشاغل المتعلقة بخطر الكوارث في دورة صياغة المشاريع

مادلين س.ثومسن ,أنجيل ج. مونيوز , ريمي كزن و جوي شوميك غليموت

الملخص

الخلفية: خلال الثلاثين عامًا الماضية ، أدى تطور نظم المعلومات الجغرافية والأقمار الصناعية لمراقبة الأرض إلى إحراز تقدم مهم في مراقبة عوامل الطقس ، المناخ ، العوامل البيئية والعوامل ذات المنشأ البشري التي تؤثر على الحد من أو عودة ظهور الأمراض المحمولة بالنواقل. أدَّت التحليلات الناتجة عن الجمع بين نظم المعلومات الجغرافية (GIS) والاستشعار عن بعد إلى تحسين معرفة العوامل المناخية والبيئية و عوامل التنوع البيولوجي التي تؤثر على الأمراض المحمولة بالنواقل (VBDs) مثل الملاريا وداء الليشمانيا الحشوية وحمى الضنك وحمى الوادي المتصدع وداء البلهارسيا ومرض شاغاس المرض و داء اللولبيات. ساعدت هذه المعرفة والمنتجات التي تم تطويرها باستخدام بيانات يتم استشعارها عن بعد ومساعدة صناع القرار على تخصيص موارد محدودة بشكل أفضل في مكافحة الأمراض المحمولة بالنواقل(VBDs).

الجسم الرئيسي: لأن الأمراض المحمولة بالنواقل (VBDs) ترتبط بالمناخ والبيئة ، نقدم هنا تجربتنا خلال الـ 4 سنوات الماضية في العمل مع المشاريع في إطار ، منظمة الصحة العالمية (WHO) / البرنامج الخاص للبحوث والتدريب في أمراض المناطق المدارية (TDR) - مبادرة مركز أبحاث التنمية الدولية (IDRC) حول الأمراض المحمولة بالنواقل (VBDs) وتغير المناخ لدمج المعلومات المناخية والبيئية في عمليات البحث وصنع القرار. تعرض الأقسام التالية المنهجية التي طورناها ، والتي تستخدم الاستشعار عن بعد لرصد تقلبات المناخ ، والظروف البيئية ، وتأثيراتها على ديناميات الأمراض المعدية. ثم نوضح كيف يمكن الوصول إلى البيانات التي تم استشعارها عن بعد وتقييمها وكيف يمكن دمجها في عمليات البحث وصنع القرار من أجل رسم خرائط المخاطر ، وإنشاء أنظمة الإنذار المبكر ، باستخدام مثالين من مشاريع منظمة الصحة العالمية الخاصة بتقارير الأمراض المزمنة (TDR) المستندة إلى تحليل البلهارسيا في جنوب إفريقيا والتريبانوزوما في تنزانيا.

الاستنتاجات: تم استخدام الأدوات المقدمة في هذا المقال بنجاح من قبل المشاريع في إطار مبادرة أبحاث IDRC-WHO / TDR حول VBDs وتغير المناخ . إلى جانب بناء القدرات ، فإنها تمثل عملاً مهماً يمكن أن يساهم بشكل كبير في: 1) أهداف استجابة منظمة الصحة العالمية العالمية لمكافحة ناقلات الأمراض و 2) أهداف التنمية المستدامة خاصةً تلك المتعلقة بالصحة والعمل المناخي.

Translated from English version into Arabic by Free bird, proofread by Abdessalam AIT TOUIJAR, through

将气候和环境信息纳入公共卫生的方法和工具

Pietro Ceccato, Bernadette Ramirez, Tawanda Manyangadze, Paul Gwakisa, Madeleine C. Thomson

摘要:

引言：在过去30年中，地理信息系统（GIS）和地球观测卫星的发展在监测影响媒传疾病（VBDs）发病率下降或再现的天气、气候、环境和人为因素方面取得了重要进展。GIS和遥感相结合进行分析提高了对影响疟疾、内脏利什曼病、登革热、裂谷热、血吸虫病、美洲锥虫病和钩端螺旋体病等VBDs的气候、环境和生物多样性因素的认识。利用遥感探测技术开发的这些

内容和产品有助于并将持续帮助决策者更好地分配有限的资源以对抗VBDs。
正文：由于VBDs同气候和环境密切相关，本文展示了在过去4年中我们与世界卫生组织（WHO）热带病培训研究特别规划署（TDR）-国际发展研究中心（IDRC）合作的关于将气候和环境信息纳入研究和决策过程的项目。以下部分展示了我们开发的使用遥感监测气候改变、环境因素及其对传染病动态影响的方法。最后，我们基于南非血吸虫病分析和坦桑尼亚锥虫病分析的两个WHO-TDR项目展示如何访问和评估遥感数据，以及如何将这些数据纳入到绘制风险、创建早期预警系统的研究和决策过程中。
结论：本文介绍的工具已成功应用于 WHO/TDR-IDRC 主导的 VBDs 和气候变化研究项目中。结合能力建设工作，作为工作的重要组成部分，它们可以显著推动 WHO 全球媒介控制响应目标和可持续发展目标尤其是涉及健康和气候问题的完成。

Translated from English version into Chinese by Peng Song, edited by Pin Yang

CHINESE CENTER FOR DISEASE CONTROL AND PREVENTION
NATIONAL INSTITUTE OF PARASITIC DISEASES

Données et outils en vue d'inclure les informations relatives au climat et à l'environnement à la santé publique.

Pietro Ceccato, Bernadette Ramirez, Tawanda Manyangadze, Paul Gwakisa, Madeleine C. Thomson

Résumé

Contexte: Au cours des trente dernières années, l'évolution des systèmes d'information géographique et des satellites pour l'observation de la Terre ont permis d'importants progrès dans le domaine de la surveillance des facteurs météorologiques, climatiques, environnementaux et anthropogènes qui influent sur la disparition et la réapparition des maladies à transmission vectorielle. Les analyses résultant de l'association des systèmes d'information géographique (SIG) à la télédétection ont amélioré notre connaissance des facteurs climatiques et environnementaux et des facteurs de biodiversité qui influencent les maladies à transmission vectorielle (MTV) telles que le paludisme, la leishmaniose viscérale, la dengue, la fièvre de la vallée du Rift, la schistosomiase, la maladie de Chagas et la leptospirose. Ces informations et les traitements développés grâce aux données obtenues par télédétection ont aidé (et aident encore) les instances décisionnaires à répartir du mieux possible le peu de ressources disponibles dans la lutte contre les MTV.
Discussion: Étant donné que les MTV sont liées au climat et à l'environnement, nous présentons ici notre expérience, menée durant ces quatre dernières années dans le cadre des projets de l'Organisation mondiale de la santé (OMS) / Le programme spécial de recherche et de formation concernant les maladies tropicales (TDR) - le Centre de Recherches pour le Développement International (CRDI) et du projet de recherche en matière de MTV et de réchauffement climatique afin d'incorporer les données climatiques et environnementales à la recherche et au processus décisionnel. Les sections ci-après exposent la méthodologie que nous avons développée, basée sur la télédétection en vue de contrôler les variations climatiques, les conditions environnementales et leurs répercussions sur l'évolution des

maladies infectieuses. Ensuite, nous présentons comment accéder aux données recueillies par télédétection, comment les analyser et comment les intégrer à la recherche et au processus décisionnel afin de cartographier les risques et mettre en place des dispositifs d'alerte rapide en utilisant deux exemples provenant des projets de recherche sur les maladies tropicales de l'OMS basés sur l'étude de la schistosomiase en Afrique du Sud et de la trypanosomiase en Tanzanie.
Conclusions: Les instruments présentés dans cet article ont été utilisés avec succès dans le cadre des projets relevant de l'OMS/TDR - Projets de recherches du CRDI sur les MTV et le réchauffement climatique. Combinés au renforcement des compétences, ils représentent un travail considérable, capable de contribuer de manière significative à la lutte antivectorielle mondiale menée par l'OMS et aux objectifs de développement durable, notamment ceux en matière de santé et d'action écologique.

Translated from English version into French by William Squire, proofread by Eve Anderson, edited by Pietro Ceccato, through

TRANSLATORS
WITHOUT BORDERS

Данные и инструментарий для интеграции климатической и экологической информации в общественное здравоохранение

Пьетро Чеккато, Бернадетт Рамирес, Таванда Манянгадзе, Пол Гвакиса, Мадлен К. Томсон

Аннотация
История вопроса: За последние 30 лет развитие географических информационных систем и спутников для наблюдения за Землёй обеспечили важный прогресс в наблюдении за погодными, климатическими, экологическими и антропогенными факторами, влияющими на уменьшение или возобновление трансмиссивных заболеваний. Результаты, полученные при сочетании исследований географических информационных систем (ГИС) и дистанционного зондирования, улучшили знания о климатических и экологических факторах, а также факторах биологического разнообразия, влияющих на трансмиссивные болезни, такие как малярия, висцеральный лейшманиоз, лихорадка денге, лихорадка Рифт-Валли, шистосомоз, болезнь Шагаса и лептоспироз. Эти знания и продукты, разработанные с использованием данных дистанционного зондирования, помогли и продолжают помогать специалистам, принимающим решения, лучше распределять ограниченные ресурсы в борьбе с трансмиссивными болезнями.
Основная часть: Поскольку трансмиссивные болезни связаны с климатом и окружающей средой, здесь представлен наш опыт последних 4 лет, в течение которых с целью интеграции климатической и экологической информации в исследования и процессы принятия решений мы работали над проектами под эгидой Всемирной организацией здравоохранения (ВОЗ) — Исследовательской инициативы по трансмиссивным болезням и изменению климата, Специальной программы по научным исследованиям и подготовке специалистов в области тропических болезней (программа TDR), а также Международного научно-исследовательского

центра развития (IDRC). В следующих разделах представлена разработанная нами методология, которая использует дистанционное зондирование для мониторинга изменчивости климата, условий окружающей среды и их воздействия на динамику инфекционных заболеваний. Далее демонстрируется способ получения доступа и оценки данных дистанционного зондирования, а также способ их интеграции в исследования и процессы принятия решений для картирования рисков и создания систем раннего оповещения, используя два примера из проектов TDR ВОЗ, основанных на исследовании шистосомоза в Южной Африке и трипаносомоза в Танзании.

Выводы: Инструментарий, представленный в этой статье, был успешно использован в проектах под эгидой ВОЗ — Исследовательская инициатива TDR-IDRC по трансмиссивным болезням и изменению климата. В сочетании с наращиванием потенциала, они являются важной частью работы, которая может в значительной степени способствовать: 1) целям проекта ВОЗ Глобальные меры по борьбе с переносчиками инфекции и 2) Целям устойчивого развития, особенно в области здравоохранения и климата.

Translated from English version into Russian by Daria, proofread by Alexander Somin, through

TRANSLATORS
WITHOUT BORDERS

Datos y herramientas para la integración de la información medioambiental y climática en la salud pública

Pietro Ceccato, Bernadette Ramirez, Tawanda Manyangadze, Paul Gwakisa, Madeleine C. Thomson

Resumen

Antecedentes: En los últimos 30 años, el desarrollo de los sistemas de información geográfica y de satélites para la observación de la Tierra han posibilitado importantes avances en la monitorización de los factores meteorológicos, climatológicos, ambientales y antropogénicos que influyen en la disminución o el repunte de las enfermedades transmitidas por vectores. Mediante análisis que integran los sistemas de información geográfica (SIG) y la teledetección se ha mejorado el conocimiento de los factores climáticos, ambientales y bióticos que inciden en las enfermedades transmitidas por vectores (ETV) como la malaria, la leishmaniasis visceral, el dengue, la fiebre del Valle del Rift, la esquistosomiasis, la enfermedad de Chagas y la leptospirosis. Este conocimiento y los productos desarrollados utilizando datos teledetectados han ayudado y continúan ayudando a los responsables de tomar decisiones a asignar mejor los recursos limitados en la lucha contra las ETV.

Cuerpo principal: Dado que las ETV están relacionadas con el clima y el medio ambiente, presentamos aquí nuestra experiencia de los últimos cuatro años trabajando en el marco del Programa Especial de Investigaciones y Enseñanzas sobre Enfermedades Tropicales (TDR) de la Organización Mundial de la Salud (OMS) - Iniciativa de Investigación de ETV y Cambio Climático del International Development Research Centre (IDRC) para incorporar la información climática y medioambiental en la investigación y los procesos de toma de decisiones. Los siguientes apartados presentan la metodología

que hemos elaborado, que utiliza la teledetección para monitorizar la variabilidad climática y las condiciones medioambientales así como su impacto en la dinámica de las enfermedades infecciosas. Luego mostramos cómo se puede acceder y evaluar los datos teledetectados y cómo se pueden incorporar a la investigación y a los procesos de toma de decisiones para crear mapas de riesgos y sistemas de alerta temprana utilizando dos ejemplos tomados de proyectos de investigación en enfermedades tropicales de la OMS dedicados al estudio de la esquisotomiasis en Sudáfrica y la tripanosomiasis en Tanzania.

Conclusiones: Las herramientas presentadas en este artículo se han utilizado con éxito en los proyectos enmarcados en la TDR de la OMS / la Iniciativa de Investigación de ETV y Cambio Climático del IDRC. En combinación con el fortalecimiento de capacidades, constituyen un instrumento importante que puede contribuir de manera significativa a: 1) los objetivos de la Respuesta Mundial para el Control de Vectores de la OMS y 2) los Objetivos del Desarrollo Sostenible, especialmente los relacionados con la salud y la acción por el clima.

Translated from English version into Spanish by Ribcamar, proofread by Felipe Chávez, through

TRANSLATORS
WITHOUT BORDERS
